# Development and application of coupled system dynamics and game theory: A dynamic water conflict resolution method

**DOI:** 10.1371/journal.pone.0188489

**Published:** 2017-12-07

**Authors:** Mehdi Zomorodian, Sai Hin Lai, Mehran Homayounfar, Shaliza Ibrahim, Gareth Pender

**Affiliations:** 1 Department of Civil Engineering, Faculty of Engineering, University of Malaya, Kuala Lumpur, Wilayah Persekutuan Kuala Lumpur, Malaysia; 2 Department of Agricultural and Biological Engineering, University of Florida, Gainesville, United States; 3 School of Energy, Geoscience, Infrastructure and Society, Heriot-Watt University, Edinburgh, Scotland, United Kingdom; Bristol University/Remote Sensing Solutions Inc., UNITED STATES

## Abstract

Conflicts over water resources can be highly dynamic and complex due to the various factors which can affect such systems, including economic, engineering, social, hydrologic, environmental and even political, as well as the inherent uncertainty involved in many of these factors. Furthermore, the conflicting behavior, preferences and goals of stakeholders can often make such conflicts even more challenging. While many game models, both cooperative and non-cooperative, have been suggested to deal with problems over utilizing and sharing water resources, most of these are based on a static viewpoint of demand points during optimization procedures. Moreover, such models are usually developed for a single reservoir system, and so are not really suitable for application to an integrated decision support system involving more than one reservoir. This paper outlines a coupled simulation-optimization modeling method based on a combination of system dynamics (SD) and game theory (GT). The method harnesses SD to capture the dynamic behavior of the water system, utilizing feedback loops between the system components in the course of the simulation. In addition, it uses GT concepts, including pure-strategy and mixed-strategy games as well as the Nash Bargaining Solution (NBS) method, to find the optimum allocation decisions over available water in the system. To test the capability of the proposed method to resolve multi-reservoir and multi-objective conflicts, two different deterministic simulation-optimization models with increasing levels of complexity were developed for the Langat River basin in Malaysia. The later is a strategic water catchment that has a range of different stakeholders and managerial bodies, which are however willing to cooperate in order to avoid unmet demand. In our first model, all water users play a dynamic pure-strategy game. The second model then adds in dynamic behaviors to reservoirs to factor in inflow uncertainty and adjust the strategies for the reservoirs using the mixed-strategy game and Markov chain methods. The two models were then evaluated against three performance indices: Reliability, Resilience and Vulnerability (R-R-V). The results showed that, while both models were well capable of dealing with conflict resolution over water resources in the Langat River basin, the second model achieved a substantially improved performance through its ability to deal with dynamicity, complexity and uncertainty in the river system.

## Introduction

In recent decades, the allocation of water resources has become more complicated and controversial due to the various factors involved in the dynamics of a water system, the effects of human activities, socio-economic changes, climate change, environmental considerations, sustainable development concerns, and changes in hydrological and hydraulic conditions. The shortage of available resources and the dramatic growth in demand in different sectors have made water resources an important source of conflicts. In this increasingly complex situation, decision makers more and more require reliable models to allocate water resources between different stakeholders effectively and efficiently in order to avoid such conflicts [1–5].

Numerous scholars have applied various techniques in a bid to find the best way of making such allocation decisions [6]. A range of different simulation models have been suggested to develop both negotiation support [7] and decision support systems [8–13]. In addition, various optimization techniques, such as Linear Programming (LP) [14], Non-Linear Programming (NLP) [15], Dynamic Programming (DP) [16], Stocastic Dynamic Programming (SDP) [17–20], Optimal Control Theory (OCT) [21, 22], Artificial Intelligence (AI) [23, 24], and Game Theory (GT) [25–28], have been suggested as a means of solving allocation problems. However, both simulation and optimization methods have not only advantages, but also substantial drawbacks.

Modeling human behaviors as a highly dynamic component in water resources systems is a particularly challenging task; yet finding a method to accurately take account of the many possible objectives and resulting behaviors of stakeholders is crucially important to finding a sustainable resolution. Most conventional methods try to convert the behavior, preferences and goals of the parties involved into one objective function using economic elements. However, aggregating multiple and often conflicting goals in this way is almost always impossible due to the complexity involved in water resource systems [29–31].

This is where game theory comes in. Game theory facilitates the modeling of human interactions and behaviors within water resources systems, and has consequently been suggested and applied to allocation problems by many researchers [32–35]. An allocation problem can be considered as a cooperative or non-cooperative bargaining game. In this context, the Nash Bargaining Solution (NBS) can uniquely and efficiently provide solutions for resource allocations among competing players [36–39]. While many models have been developed based on this method, some issues limit the application of these models. One such common issue is that many of these models have been developed for a single reservoir system and cannot be easily adapted for use in multi-reservoir systems, mainly due to the “curse of dimensionality” (see [17, 27, 28, 34, 40, 41]). Another issue is that many of these solutions do not consider the dynamicity of supply or demand components during optimization processes (cf [25, 26]). Such models tend to take a static view of demand points while seeking to develop the optimum operation rules. However, optimized operation policies developed on this basis can fail to optimally allocate water as they are not capable of adjusting policies according to dynamic changes in demand points over time.

In practice, conflicts over water resources are often highly dynamic, especially in fast developing areas; moreover, many water resource systems rely on multi-reservoir systems. Thus, recognizing how a water allocation problem and related factors evolve over different stages is crucial to avoid misinterpretions or wrong decisions [31]. Dynamic games have generally been suggested to deal with this issue. However, the fast changing dynamics of current water resource systems require a modeling system that is capable of updating the behavior, preferences and goals of players, as well as external factors which affect the whole system or the games involved, so it can more accurately represent and reflect what is actually happening. Simulation-optimization (SO) models are one possible way of achieving this. As Singh [42] suggested, “*it is unlikely to get an appropriate solution with simulation or optimization techniques alone*, *and thus the combined use of simulation and optimization models is essential*.”

Among different simulation approaches, a system approach can provide stakeholders and decision makers with a systematic decision making process by improving their understanding of the problem, as well as of the consequences of their decisions and actions on the resulting outcomes, in ways that can help them see and achieve new sustainable solutions for a conflict [43, 44]. Specifically, as a method for operationalizing systems thinking, system dynamics (SD) can facilitate a more holistic understanding of water resource systems as well as strategic decision making. It can also facilitate system modeling, and produce a better analysis of a system’s behavioral trends, utilizing feedback loops [45–48]. The main advantages of SD over other simulation methods are the transparency of the models involved and the presence of feedback loops, both of which help models to keep track of fast changing parameters in the system [48, 49].

In sum, developing models using a combination of SD and GT can help to reassure decision makers that the final decision is the optimal one, and one which takes full account of the dynamics of physical, environmental, financial, political and social aspects.

In line with the above, in this study we have sought to demonstrate the advantages of coupling system dynamics and game theory as a mathematical tool for dynamic conflict resolution. While some scholars have proposed complicated and complex SD models to simulate the interactions between supply and demand sub-systems, we have employed two simple integrated water resource allocation models in this study to demonstrate how this methodology can manage efficiently different levels of complexity in a multi-reservoir system. Moreover, this study tests and illustrates the application of the suggested method, through these two models, to a specific catchment area (Langat River basin). The first model attempts to manage water between two demand sites and two reservoirs without taking into account the hydrological uncertainty of inflows into the reservoirs. The second model, on the other hand, also takes into account the hydrological uncertainty of inflows in managing the reservoirs, using a combination of mixed-strategy games and the Markov chain method. Both models seek to simulate the dynamic behaviors of demand and supply components, and at the same time optimize water allocation to the demand sites and its release from reservoirs at every step along the time horizon. To develop these models, all the classes and routines were coded in MATLAB. Both models were then run seasonally for twenty-two years. Finally, their performances were evaluated of this period by selected performance indices.

## Methodology

### Conflict over water resources in a multi-reservoir system: problem statement

Based on a dynamic view of a bargaining game, a general approach to conflict resolution in water allocation can be stated as Eq 1. The main aim here is to optimize the overall utilities (*Ū*_*t*_) of water users resulting from the water allocation vector (x) and subject to a constraint on available water at time *t*.

$$M_{t}={Max}_{x_{1},\ldots ,x_{n}}{\bar{U}}_{t}(X_{t}),\;Subject\;to:\sum_{j=1}^{n}x_{j}\leq S_{t}+I_{t}$$ (1)

where *M*_*t*_ is the indirect utility at time *t*, illustrating the maximized level of satisfaction for any given time *t*. *X*_*t*_ *= {x*_*j*_, *j = 1*, *…*, *n}* is the allocated water vector to “n” different water users (including demand sites and reservoir operators), while the overall utility of the water allocated is represented by *Ū*_*t*_ *(X*_*t*_*)*. Also, *S*_*t*_ is reservoir storage at the start of time *t*, and *I*_*t*_ is the inflow discharge during time *t*.

The main aim of this study is to develop a methodology for finding *M*_*t*_ that takes into account the dynamicity and uncertainty of the supply and demand elements, and at the same time can be readily applied to multi-reservoir systems.

### Development of a dynamic conflict resolution method based on a combination of system dynamics (SD) and game theory (GT)

Bargaining games are used to simulate situations in which two or more consumers (players) compete to get an optimum share from a proportion of limited resources. The commonly known Nash Bargaining Solution (Eqs 2–4) is widely used to simulate these interactions in the context of the allocation of a limited water supply. In this study, a dynamic form of these equations is employed, so that the dynamicity of the behavior of water users can be taken into account during the optimization process. Specifically, this study uses the Nash bargaining solution as part of a proposed iterative solution to solve the dynamic game model (Eq 1), using a simulated annealing approach as an optimization technique [40]. Nash Jr [50] proposed a solution to a two-person cooperative game which is more robust than most alternative methods, such as weighing techniques [40]. The generalized version of this for n-person cases, developed by Harsanyi [51], and Harsanyi and Selten [52] and represented by Chae and Heidhues [38] in Eqs 2–4 below, is used in this study.

$$Maximize\;Z=(U_{1}-d_{1})(U_{2}-d_{2})\ldots (U_{n}-d_{n})$$ (2)

$$Subject\;to\;U_{i}\geq d_{i},\;i=1,2,\ldots ,n$$ (3)

$$\bar{U}=(U_{1},U_{2},\ldots ,U_{n})\in \{y\in R^{N}:\;y_{i}=f_{i}(x),\;x\in X\;\forall i\in N\}$$ (4)

where *U*_*i*_ and *d*_*i*_ refer to the utility function enjoyed by players and their utility at the disagreement point; *N = {1*, *…*, *n}* represents a group of players; *X = {(x*_*1*_, *x*_*2*_, *…*, *x*_*n*_*)*: *x*_*n*_ *ϵ ℝ}* is the decision space; and *f*_*i*_*(x)* is the payoff vector.

### Combining system dynamics and the Nash Bargaining Solution

As the main water users (players) are demand sites and reservoirs in water systems, a dynamic utility function is employed for each of these components. To find the actual values of these, the functions were estimated based on the characters of the players and from questionnaires as reported in the Utility Development section. Hereafter in this text, we use “*satisfaction*” instead of “*utility*” to describe this element, due to our slightly different definition of utility which will be explained later.

In the case of Demand Site Saatisfaction Functions, Madani and Mariño [45] suggested that utility should refer to the total satisfaction of users from the available water in the basin. While this definition is useful, it is based on a static view of players’ utility. In this study, the authors have modified this definition in order to add in dynamicity: utility then becomes the total satisfaction of users based on the percentage of their demand met by the supplied water. Using this definition, DSSF becomes a dynamic function which is updated at every step in the modeling process based not only on the allocated supply, but also on demand: *DSSF*_*t*_ *(x*_*t*_, *d*_*t*_*)*.

To take dynamicity into account in the case of Reservoir Satisfaction Fanuction, Ganji & Khalili [40] suggested that utility should refer to the total level of satisfaction of the reservoir operator with the ratio of available storage compared to the maximum potential storage in the reservoir. It follows from this definition that the satisfaction function depends on the amount of release and inflow to the reservoir at time *t*, and its coefficients update at every time step: *RSF*_*t*_ *(R*_*t*_, *I*_*t*_*)*.

### Improving reservoir management using the Markov chain method and mixed-strategy games

Taking a strategy *s*_*i*_ for player *i* as any probability distribution over the actions set of *A*_*i*_, a game is a pure strategy game when only one action is played with positive probability, while it is a mixed strategy game when more than one action is played with positive probability. The utility function definition for mixed strategy games is presented in Eqs 5 and 6 below [53].

$$u_{i}(s)=\sum_{a\in A}Pr(a|s)\;u_{i}(a)$$ (5)

$$\mathrm{Pr}\left(a|s\right)=\prod_{j\in N}s_{j}(a_{j})\;and\;a=(a1,a2,\ldots ,an)\;\epsilon \;Aj$$ (6)

In this study, it is assumed that demand sites play a pure strategy game. Specifically, each demand site is assumed to have one strategy by which it tries to maximize its satisfaction level by securing its total demand. However, in order to factor in the effects of inflow uncertainty on the management of the reservoirs, the latter are assumed to adopt different behaviors in Model 1 and Model 2 respectively. In Model 1, the reservoirs play a pure strategy game, like the demand sites. In other words, they follow a similar management strategy throughout all the seasons, regardless of differences in water inflow levels between seasons.

The problem with the above approach is that, in a fast and often radically changing meteorological environment like a tropical climate, having a fixed strategy to manage a reservoir may result in system failures, or lead to more intense failures. To avoid this, in Model 2 reservoirs benefit from a coupled mixed-strategy game and Markov chain method to manage their storage based on the expected inflow at time *t+1*. To calculate the transition matrix, three inflow categories have been set: Low, Normal and High. Using the transition matrix and Eqs 5 and 6, the satisfaction function for reservoirs can be calculated as follows:

$${RSF}_{t}(R_{t},I_{t})=\left\{ \begin{array}{cc} {\bar{U}}_{s}^{L}(R_{t},I_{t}) & if\;I_{t}\in L\\ {\bar{U}}_{s}^{N}(R_{t},I_{t}) & if\;I_{t}\in N\\ {\bar{U}}_{s}^{H}(R_{t},I_{t}) & if\;I_{t}\in H \end{array}\right.$$ (7)

$$\left[\begin{array}{c} {\bar{U}}_{s}^{L}\left(R_{t},I_{t}\right)\\ {\bar{U}}_{s}^{N}\left(R_{t},I_{t}\right)\\ {\bar{U}}_{s}^{H}\left(R_{t},I_{t}\right) \end{array}\right]=\left[\begin{array}{ccc} \mathrm{Pr}\left(I_{t+1}\in L|I_{t}\in L\right) & \mathrm{Pr}\left(I_{t+1}\in N|I_{t}\in L\right) & \mathrm{Pr}\left(I_{t+1}\in H|I_{t}\in L\right)\\ \mathrm{Pr}\left(I_{t+1}\in L|I_{t}\in N\right) & \mathrm{Pr}\left(I_{t+1}\in N|I_{t}\in N\right) & \mathrm{Pr}\left(I_{t+1}\in H|I_{t}\in N\right)\\ \mathrm{Pr}\left(I_{t+1}\in L|I_{t}\in H\right) & \mathrm{Pr}\left(I_{t+1}\in N|I_{t}\in N\right) & \mathrm{Pr}\left(I_{t+1}\in H|I_{t}\in H\right) \end{array}\right]\left[\begin{array}{c} U_{s}^{L}\left(R_{t},I_{t}\right)\\ U_{s}^{N}\left(R_{t},I_{t}\right)\\ U_{s}^{H}\left(R_{t},I_{t}\right) \end{array}\right]$$ (8)

where *U*_*s*_^*L*^ *(R*_*t*_, *I*_*t*_*)* is the operator satisfaction function when drought is expected at time *t+1*, *U*_*s*_^*H*^ *(R*_*t*_, *I*_*t*_*)* is the operator satisfaction function when flood is expected at time *t+1*, and *L*, *N*, and *H* stand for “Low Flow”, “Normal Flow” and “High Flow” respectively.

The above methodology should enable the management strategy and satisfaction functions of reservoirs to be dynamically adjusted at every step of the simulation-optimization process, based on the current and expected next inflows.

### Modeling flowchart

**Fig 1:**
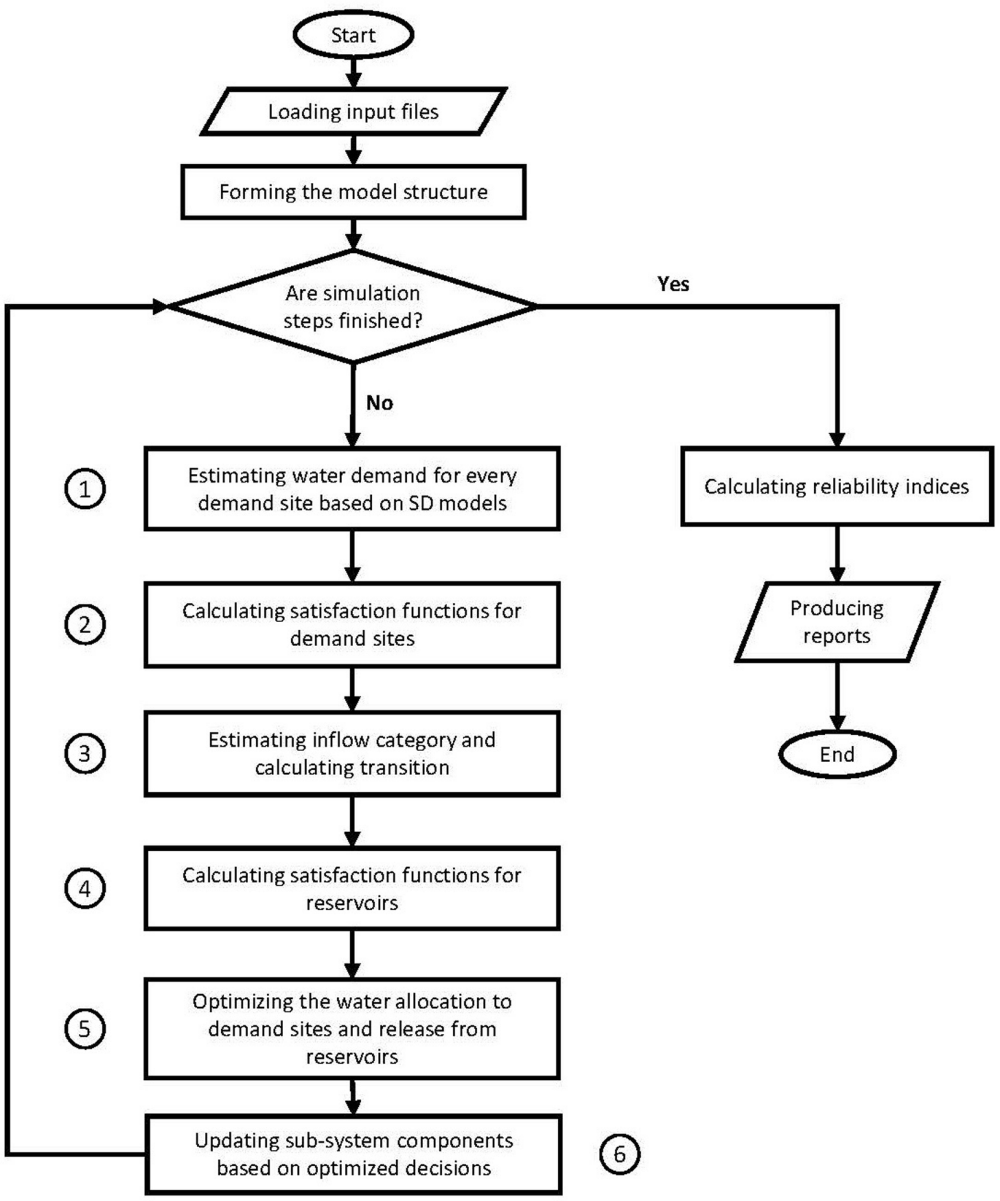
Flow chart of presented models.

In the modeling process, we initially assumed that all users and reservoir operators were fully satisfied, in order to determine the overall level of demand associated with every demand site (Fig 1, No.1). Thereafter, every DSSF was calculated (Fig 1, No.2). Next, the inflow category was determined for inflows to each reservoir, and a transition matrix was developed (Fig 1, No.3). This was followed by determining the RSF for reservoirs (Fig 1, No.4), and using the Allocation Optimizer to apply the NBS to determine the optimal storage, release levels and allocations for these (Fig 1, No.5). Finally, based on the optimal decisions resulting from the NBS, the demand site components (population growth rate, water demand per capita and their satisfaction function) and reservoir components (storage level and satisfaction function) were updated for the next time step (Fig 1, No.6).

### Structure of models

Four main types of components were the main building blocks for developing the models in this study: Demand Sites, Reservoirs, Uncontrolled Flows (Rivers and Tributaries) and an Allocation Optimizer. The first two components use the SD method to represent the dynamic behaviors of components in a real situation. The third component introduces additional water resources as an input to the model, which the model cannot control but which is available to help satisfy demand. The Allocation Optimizer meanwhile applies the Nash Bargaining Solution (NBS) to maximize system utility in a bargaining game which is dynamically played by other system components on a seasonal time frame over the years from 1992 to 2013.

### Component development

For the sake of simplicity, we first describe the building components of the models and then display the structure of the two models.

### Demand Site component

**Fig 2:**
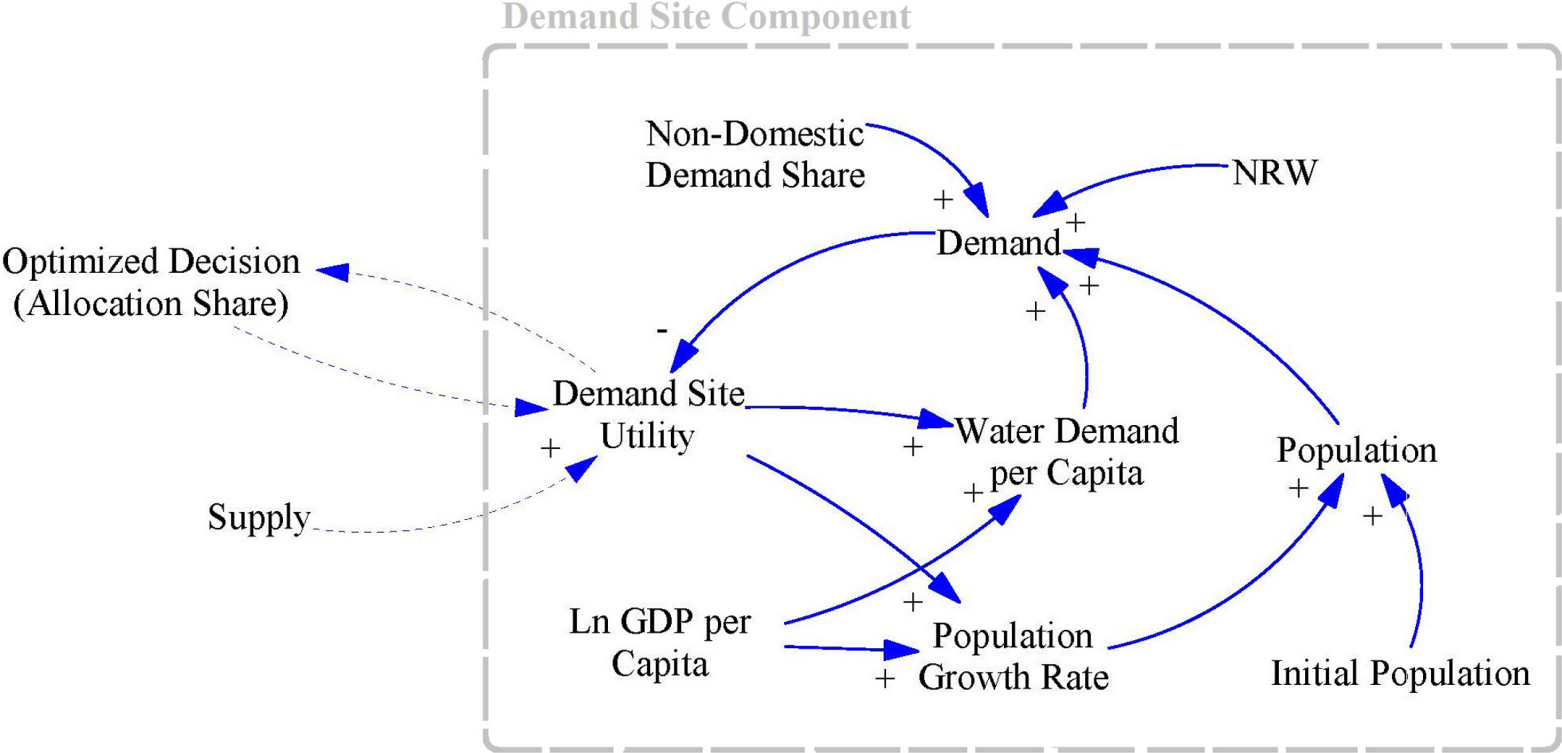
CLD for Demand Site components.

As mentioned, the Demand Site component uses the SD method to capture the dynamics of a demand site and the interactions between them. It is assumed that population growth rate and per capita water demand depend on the natural logarithm of gross domestic product (GDP) per capita, as a key economic indicator, as well as on satisfaction levels at the demand site. The conceptual model applied to Demand Site components is a modified version of the demand sub-model suggested by Madani and Mariño [45]. As a reinforcing loop, water demand per capita and population growth rates rise in line with increasing satisfaction and GDP per capita. At the same time, rises in population and water demand per capita lead to increased total water demand, which can cause unmet demand and in turn result in lower satisfaction levels in terms of supply (Balancing Loop). Fig 2 shows the Casual Loop Diagram (CLD) for the Demand Site Components.

Eqs 9–12 below were applied to quantify the conceptual model.

$$P(t)=P(ts)+\int_{ts}^{te}[PGR(t)\times P(t-1)]\;dt$$ (9)

D(t)=P(t)×WDPC(t)×[1+NRW(t)]×[1+NDDS(t)] (10)

$$WDPC(t)=\frac{U_{t}^{0.814}\times {lnGDP}_{t}^{2.623}}{1.726}+1.005$$ (11)

$$PGR(t)=\frac{U_{t}^{3.874}\times {lnGDP}_{t}^{0.748}}{0.748}+0.001$$ (12)

where *P(t)*, *PRG(t)*, *D(t)*, *WDPC(t)*, *NRW(t)*, and *NDDS(t)* are population and population growth rate, demand, water demand per capita, non-revenue water, and non-domestic demand share at time *t* respectively, and *ts* and *te* are the start and end of modeling time.

### Reservoir component

**Fig 3:**
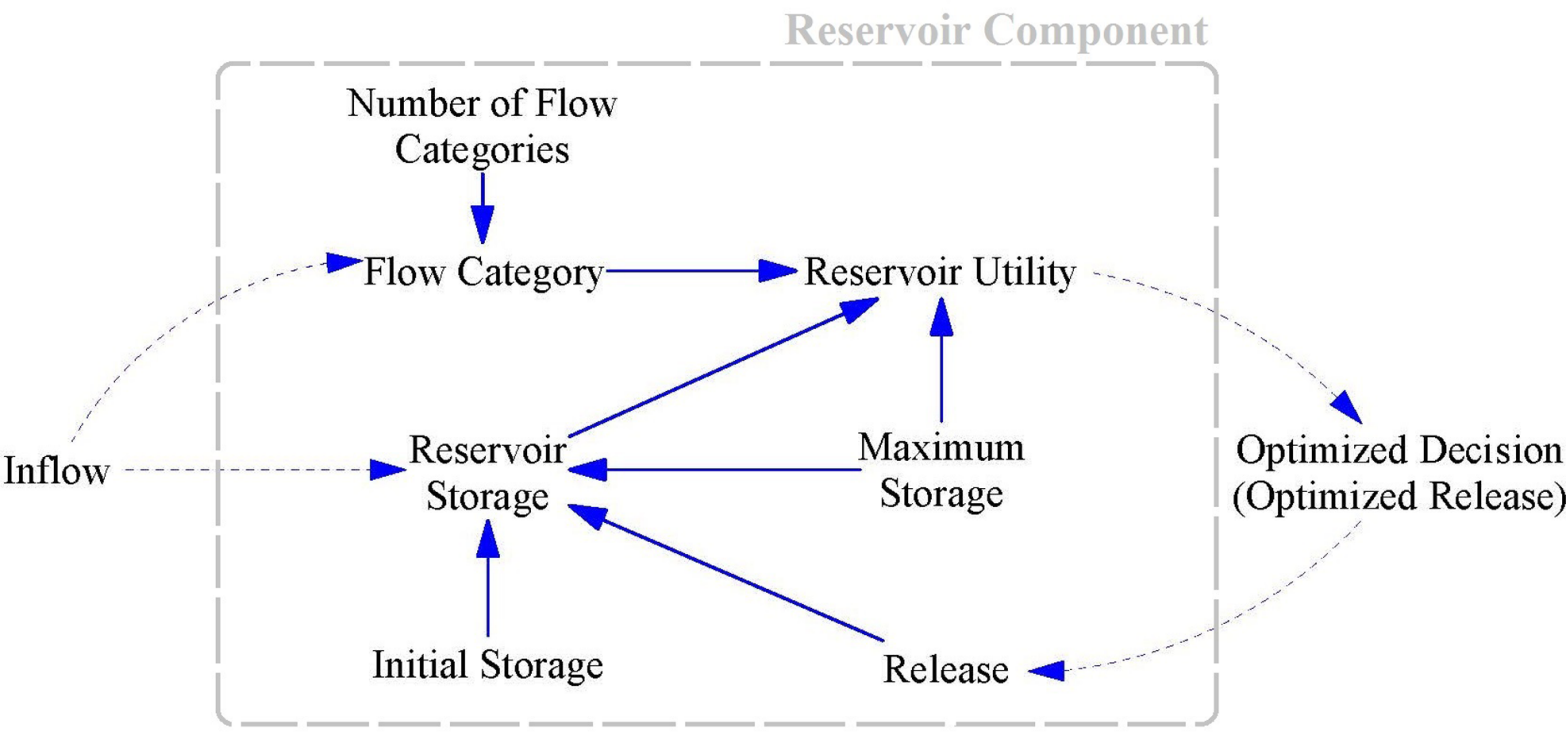
Casual Loop Diagram (CLD) for Reservoir components.

Reservoir components can simply be considered as the stock in the SD model, and can be described by a water mass balance equation. Fig 3 shows the CLD for the reservoir. As can be seen, a feedback loop exists between Reservoir Storage, Reservoir Utility, Optimized Decision and Release which allows the Allocation Optimizer component to maximize the reservoir satisfaction by adjusting the amount of water released based on a mass balance equation (Eq 13). At each time step, the Inflow, Initial Storage and Maximum Storage of the reservoir are known parameters for the model. The number of Flow Categories is equal to 1 for the first model (pure strategy game), while it is 3 for the second model (mixed-strategy game).

Eq 13 was used to convert the CLD to the Stock and Flow Diagram for the Reservoir components.

$$RS(t)=RS(ts)+\int_{ts}^{te}\left[I(t)-R(t)\right]\;dt$$ (13)

where *RS(t)*, *I(t)*, *R(t)* are reservoir storage, inflow to the reservoir and release at time *t* respectively.

### Uncontrolled flows: Rivers and tributaries

This component is a source which provides water directly to the system. If this type of component is present in a model, the amount of water provided by this component is a known parameter for the model at each time step in the modeling process. However, the model is not capable of storing such flows for the future.

### Allocation optimizer

This component utilizes the Nash Bargaining Solution (NBS) to maximize the overall satisfaction level of the whole system. The satisfaction functions added to the equations may differ depending on the availability of each component in the model. It should be noted that Uncontrolled Flow components do not have a satisfaction function. Based on both NBS and Eqs 2–13, overall satisfaction (Eq 1) can be optimized by solving Eqs 14–16:

$$Maximize\;{\bar{U}}_{t}=\prod_{i=1}^{n}{DSSF}_{i,t}(x_{i,t},d_{i,t})\times \prod_{j=1}^{m}{RSF}_{j,t}(R_{j,t},I_{j,t})$$ (14)

$$Subject\;to\;:\;x_{i,t}\leq d_{i,t},\;when\;i=1,2,\ldots ,n$$ (15)

$$s_{j}^{min}\leq s_{j,t+1}\leq s_{j}^{max},\;when\;j=1,2,\ldots ,m$$ (16)

where *Ū*_*t*_ is the Nash bargaining product indicating overall satisfaction at every step along the horizon, *DSSF* is the demand site satisfaction function, *x*_*i*_ is the amount of water allocated to each demand site, *d*_*i*_ is demand associated with each demand site, *RSF* is reservoir satisfaction function, *R*_*j*_ indicates release from reservoirs, *I*_*j*_ stands for inflow to the reservoir, *s*_*j*,*t+1*_ is available storage in reservoirs after optimization, *n* and *m* are the number of demand sites and reservoirs respectively, and *s*^*min*^ and *s*^*max*^ are minimum and maximum reservoir storages.

### Development of the models

As a first step, we considered two demand sites, two reservoirs, and two uncontrolled water supplies from tributaries to the model. Our first model seeks to maximize total satisfaction (utility) by controlling not only the allocation of water to the demand sites but also the levels of water released from the reservoirs. However, its optimizer does so step by step, without taking account of seasonal changes of inflow into the reservoirs.

**Fig 4:**
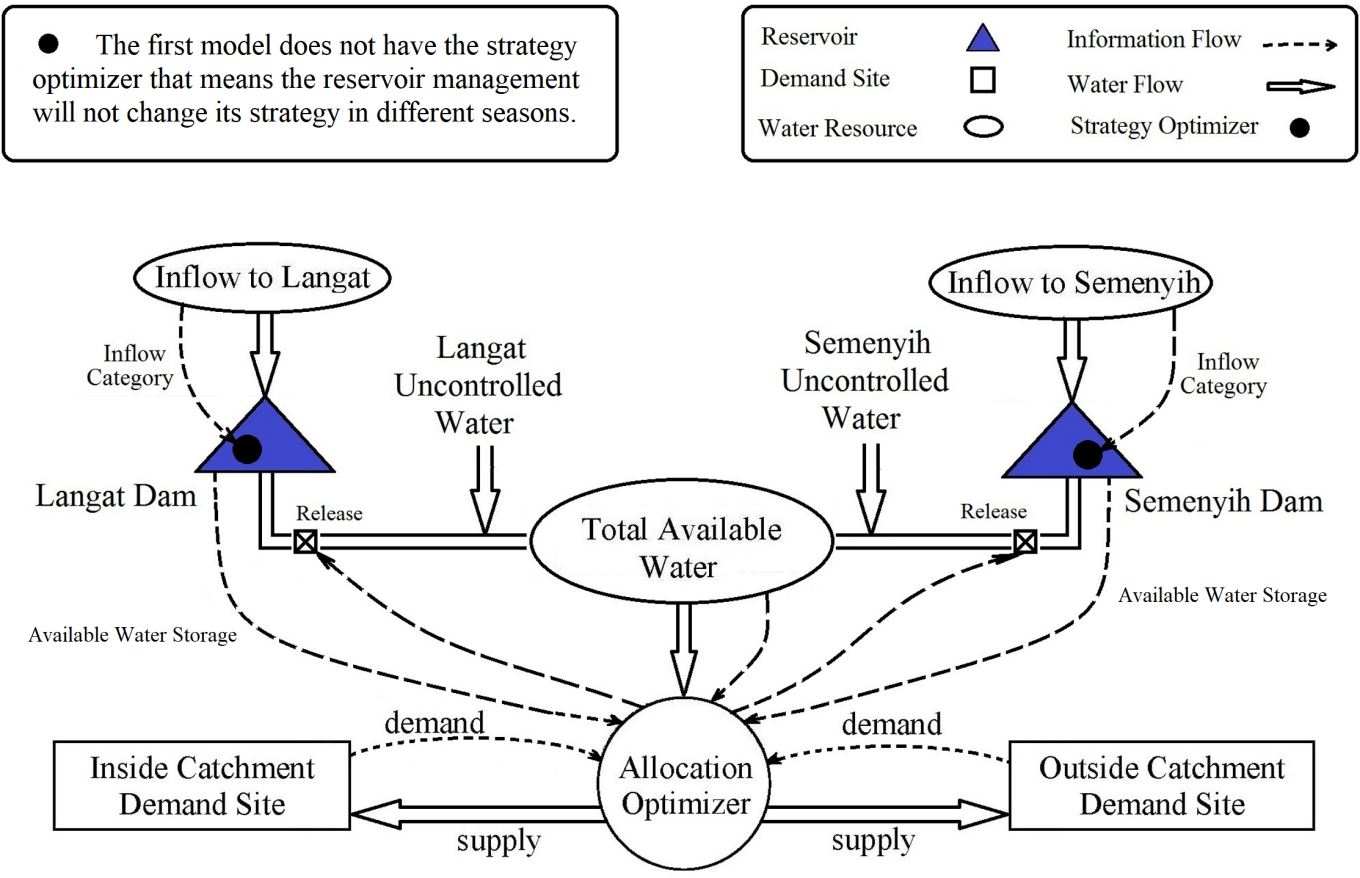
Schematic diagram of models.

In our second model, we add in a combination of the Markov chain method and mixed-strategy game theory to deal with seasonal changes in inflow into the reservoirs. In this model, reservoir operators adjust their strategies dynamically based on the expected inflow at the next time step. To achieve this, three categories of inflow, namely High Flow, Normal Flow and Low Flow, were used, and the transition matrix for each category was calculated through the Markov chain method. In addition, we developed three satisfaction functions for the reservoirs corresponding to each inflow category. Finally, we used mixed-strategy game theory (Eqs 7–8) to dynamically calculate the best response of reservoirs to both the current and expected next inflow situations for each time step, and applied these as reservoir satisfaction functions in the allocation optimizer component. Fig 4 contains a schematic diagram of the two models, showing the differences between them.

### Performance indices

In this study, three performance indices–Reliability, Resilience and Vulnerability (R-R-V)–were used to evaluate the results of the models.

### Reliability

Reliability is the capability of a system or sub-system to perform its mandatory tasks under the required conditions for a specified period of time [54]. Various reliability definitions have been applied by researchers into water resources (for example [25–27, 34, 35, 40, 41, 55–57]).

To compare the capability and efficiency of our models, we used volumetric and occurrence reliability measurements of the reservoirs and demand sites. These are explained in more detail below.

Occurrence reliability is defined as:

$$R_{n}=\left(1-\frac{Number\;of\;failures\;in\;design\;period}{Length\;of\;design\;period}\right)\times 100$$ (17)

where *R*_*n*_ is the overall component reliability. Failure is defined variously depending on the component type. For the reservoir component, failure means violating the storage limit bounds (maximum and minimum allowable storage values), while for the demand site component, it means an allocation lower than the demand in that demand site.

According to the definition of failure, the volumetric reliabilities of reservoir (R_v-s_) and demand site (R_v-d_) components are:

$$R_{v-s}=\frac{Total\;storage\;shortfall\;or\;overflow}{Total\;available\;water\;into\;reservoir\;during\;planning\;horizon}$$ (18)

$$R_{v-d}=\frac{100}{n}\sum_{i=1}^{n}\left(\frac{Supplied\;Water}{Demand}\right)$$ (19)

where *n* is the number of time steps for the planning horizon. The value of *R*_*v−s*_ can vary from 0 to values even larger than 1, with values larger than 1 indicating that the sum of the volume of failures is more than the total available water in the reservoir system during the planning horizon. A zero value indicates that there were no failures, resulting in a system volumetric reliability of 100%. Other reliability indices can vary between 0 and 100%, with values towards 100% indicating better performance by the model.

### Resilience

Resilience measures how fast a system is likely to recover and return to a satisfactory state after a failure event [54, 58]. According to Kjeldsen and Rosbjerg [58], this parameter can be explained mathematically using Eq 20:

$$Res={\left(\frac{1}{F}\;\sum_{j=1}^{F}d(j)\right)}^{-1}$$ (20)

where *F* is the total number of failure events and *d(j)* is the duration of the *j*th failure event. This index varies between 0 and 100%, with lower percentage values indicating a lower performance and hence a higher degree of risk, especially for prolonged events.

### Vulnerability

It is important to consider the potential consequences of a failure in a water distribution system, even if the chances of a failure event occurring are small [54]. Vulnerability does this, by indicating the possible damage caused by a failure if it occurs. Kjeldsen and Rosbjerg [58] suggested that the vulnerability of a system can be defined based on a definition by Hashimoto and Stedinger [54], as shown in Eq 21 below:

$$Vul=\frac{1}{F}\;\sum_{j=1}^{F}v(j)$$ (21)

where *v(j)* is the deficit volume of the *j*th failure event. A lower value for vulnerability indicates lower expected damage after any failure event. Thus values close to zero indicate a better–more resilient–performance by the model.

### Case study

**Fig 5:**
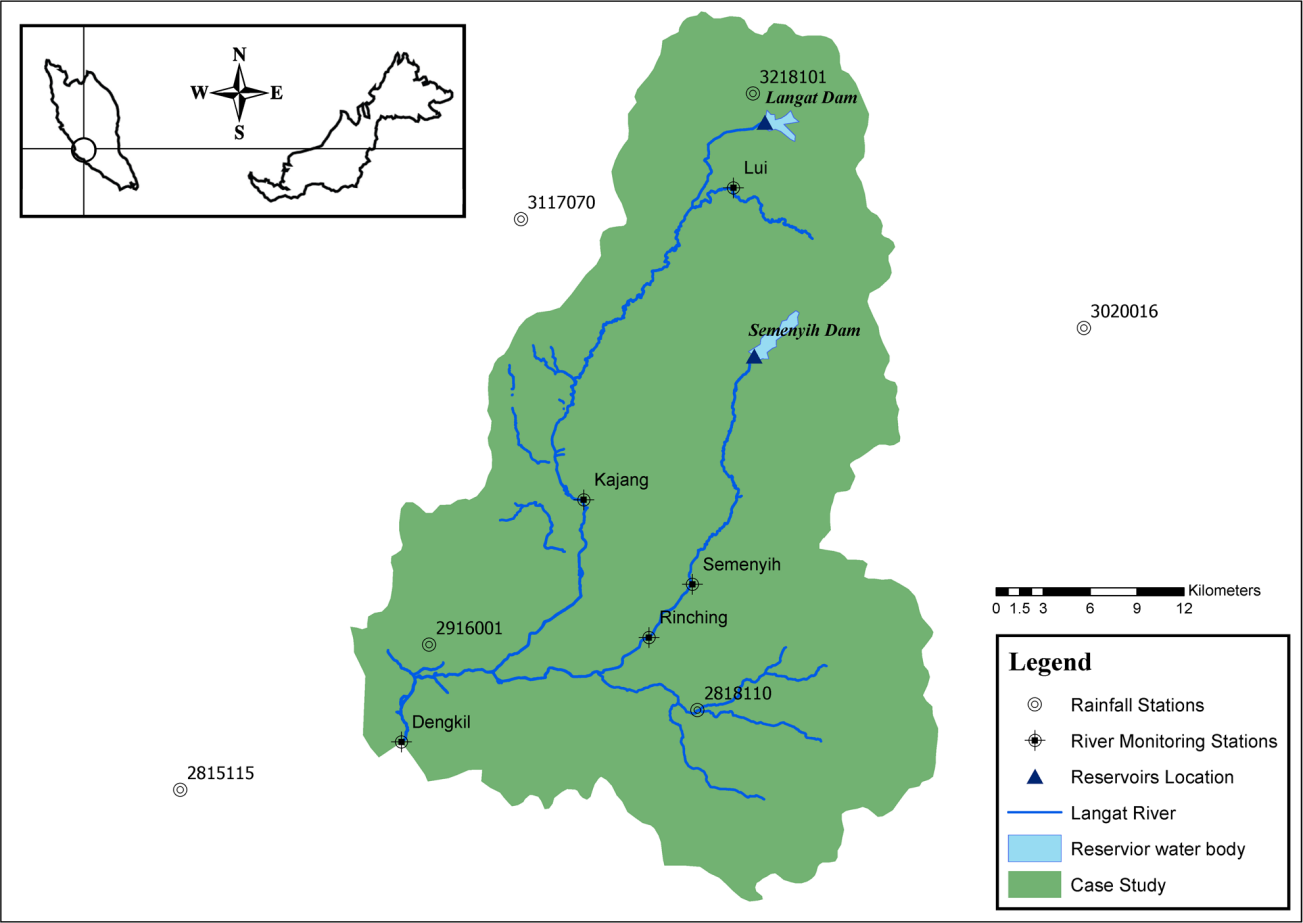
Langat River Basin with available monitoring stations.

The Langat River basin is located in the southern and south-eastern parts of the state of Selangor in Malaysia. Fig 5 below shows the boundaries of the catchment and the main tributaries of the Langat River. The basin has an area of 2350 km^2^, with a length of 141 km. It has a tropical climate, with 2145 mm of average annual rainfall and 1500 mm of annual evapotranspiration.

This study focuses on the upper Langat River basin, up to the Dengkil river monitoring station. This area has a key role in providing water for domestic and commercial usage to about 2.5 million people living inside the basin and in the surrounding area, including the capital city of Kuala Lumpur and the administrative center of Putrajaya. The catchment itself is controlled by the state government. However, it provides water to Kuala Lumpur, Putrajaya, and Cyberjaya, all of which are Federal Territories under the control of the federal government. This makes the basin a good case study for an allocation game between different stakeholders and decision makers who are willing to cooperate to solve the unmet demand problem.

## Results and discussion

### Utility development

To develop the functions which reflect the reservoir operators’ satisfaction, a questionnaire was prepared and sent to a group of experts and decision makers familiar with water issues in the catchment area. In addition, a different questionnaire was sent to students, campus staff and ordinary people to determine functions to measure the satisfaction levels of these water users (i.e. how far different levels of supply satisfied their water needs). Totally, 150 copies of both types of questionnaires were issued, in line with the guidelines by Dyer et al. [59]. These were used to quantify the preference points: both water users and water experts were asked to indicate the ranges within which their demand for water would be satisfied, and any points of disagreement. A total of 133 validly completed questionnaires were received, and were then used to estimate both kinds of satisfaction functions: RSF (reservoir satisfaction function) and DSSF (demand sites satisfaction function). We did this on the assumption that the users' judgments and answers would largely reflect the objectives and aims of their corresponding agencies. The satisfaction functions were then approximated by quadratic equations for the demand sites, and quartic equations for reservoirs, over the range of utility levels.

$$\left\{\begin{array}{ll} \;U_{d}=-0.8339\;{\left(\frac{Supply}{Demand}\right)}^{2}+1.7928\;\left(\frac{Supply}{Demand}\right)+0.036 & \;0\leq \frac{Supply}{Demand}\leq 2\\ 0 & \;Otherwise \end{array}\right\}$$ (22)

$$\left\{\begin{array}{ll} U_{s}^{L}=-7.8963x^{4}+18.611x^{3}-18.19x^{2}+8.584x-0.6017 & \;0\leq x\leq 1\\ 0 & \;Otherwise \end{array}\right\}$$ (23)

$$\left\{\begin{array}{ll} U_{s}^{N}=-6.8765x^{4}+13.753x^{3}-12.213x^{2}+5.3368x+0.0951 & \;0\leq x\leq 1\\ 0 & \;Otherwise \end{array}\right\}$$ (24)

$$\left\{\begin{array}{ll} U_{s}^{H}=-7.8963x^{4}+12.974x^{3}-9.734x^{2}+3.5471x+0.5075 & \;0\leq x\leq 1\\ 0 & \;Otherwise \end{array}\right\}$$ (25)

$$x=\frac{Reservoir\;Storage}{Reservoir\;Maximum\;Strorage}$$ (26)

Eq 22 above shows the satisfaction function for the demand sites. Eqs 23–26 represent the resulting satisfaction functions for the reservoir operator for three different situations: Low Flow (*U*_*s*_^*L*^), Normal Flow (*U*_*s*_^*N*^) and High Flow (*U*_*s*_^*H*^). Normal flow is a situation in which a normal inflow is expected in the next time step, so operators generally seek to keep the water in the reservoirs at the normal level. Low flow means a lower than average inflow is expected in the next time step, so operators tend to seek to conserve more water in the reservoirs. Finally, high flow means that a higher than average inflow is expected in the next time step; operators therefore usually prefer to release more water to prevent flooding.

In our first model, the reservoirs always play a pure strategy game in which the best satisfaction level results from storing 50% of maximum capacity in the reservoirs (Eq 24 only). In our second model, the reservoirs use three strategies with three different RSFs (Eqs 23–26). This mixed-strategy game in our second model helps operators to adapt more flexibly in order to avoid both failures due to flooding, by storing less water in the reservoir when a high chance of flooding (high flow) is expected in the next time step; and also to prepare the reservoirs by increasing the level of stored water when a high chance of drought (low flow) is expected in the next time step.

### Model calibration

The ability of a model to simulate real water systems is assessed through calibration and validation procedures. Once these are completed, the model can be used to inform optimum decisions. The population growth rate and water use per capita need to be estimated based on existing observed data, in accordance with the CLD of demand site components. The observed data for a time period of five years (2009–2013) is used to calibrate these parameters of the demand site components.

**Fig 6:**
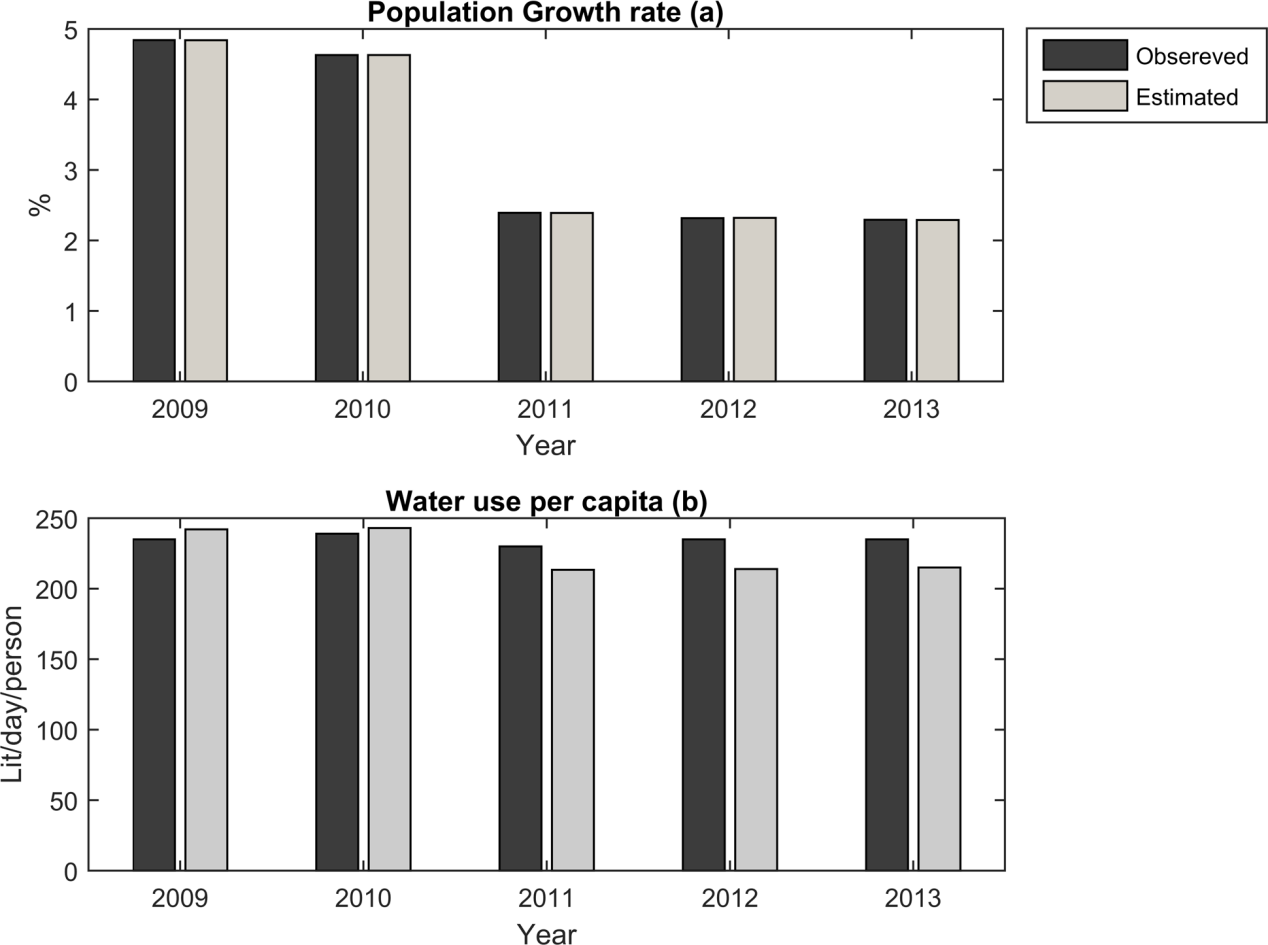
Comparison between simulated and observed parameters.

Fig 6 below shows the results of a comparison between the simulated and observed values for population growth rate and domestic water use per capita over the calibration period. Overall, the estimated parameters emerged as satisfactorily accurate compared with the actual observed data, indicating that the demand site component is acceptably calibrated to reproduce the dynamic behavior of the different parameters within this component.

### Dynamic behavior of demand site components

**Fig 7:**
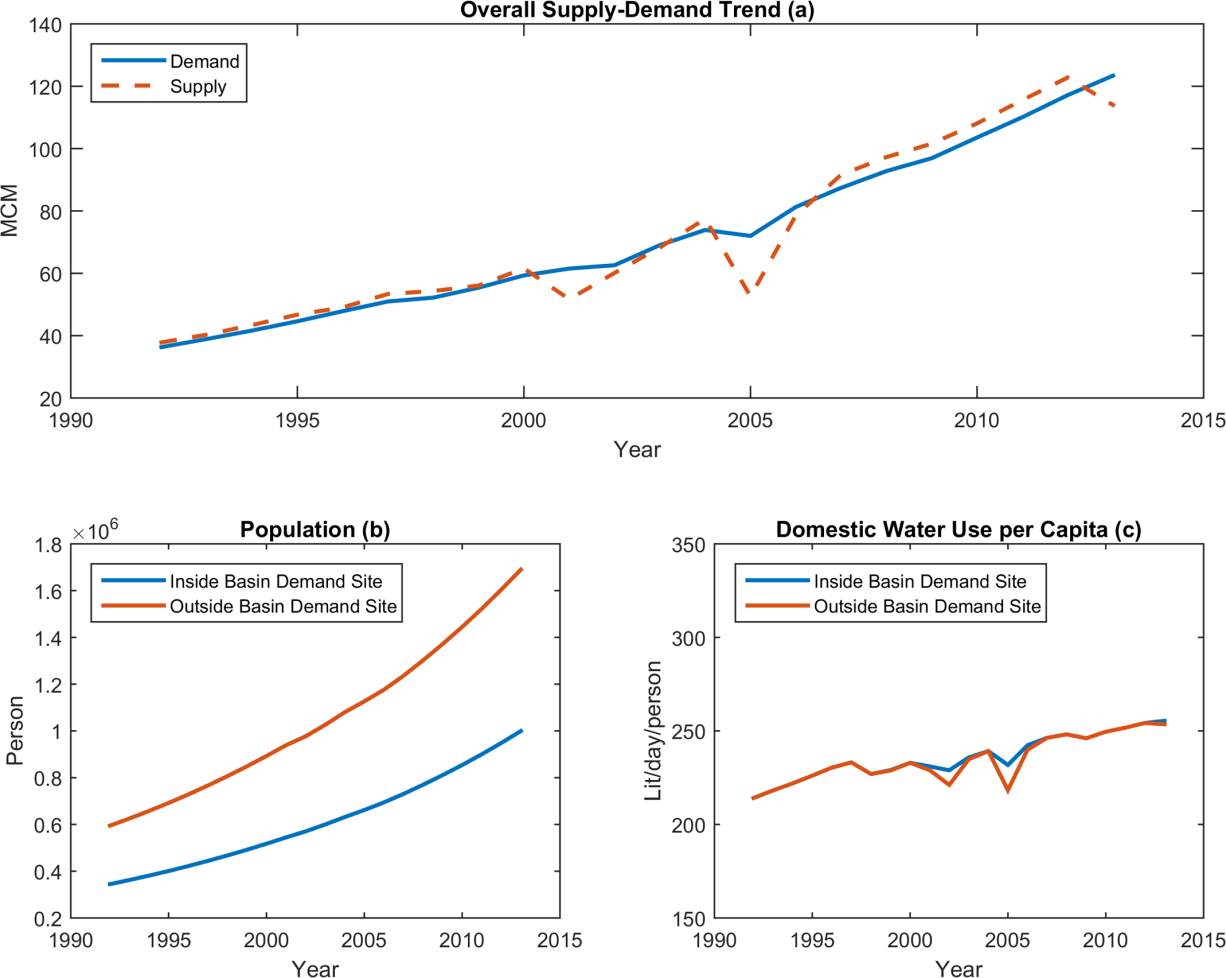
Results of system dynamics models for demand sites: (a) Overall supply-demand trend; (b) Population; (c) Domestic water usage per capita.

Fig 7 shows the modeling results of demand sites for both models. The level of demand for each site depends on economic growth and the satisfaction levels resulting from actual supply. As economic growth was the same for both models, and unmet demand during the simulation-optimization was negligible, the difference between the demand site components of the models is also negligible.

Fig 7A below shows the supply and demand trends for both models. The difference between estimated and actual demand was less than 1% at the end of simulation-optimization process. The total estimated population in the area at the end of the 22-year simulation (Fig 7B) was very close to the actual observed population of the area (both inside and outside the basin); furthermore, the total estimated population of the second model at the end of the simulation-optimization process was only about 5000 people lower than that of the first model.

Finally, Fig 7C shows the changes in domestic water use per capita during the modeling time. Again, our models estimated water usage per capita and population growth relatively accurately compared to the actual observed data. Moreover, in the worst period of drought, the models predicted that water usage per capita would fall by about 6% as the demand sites responded to the dry conditions.

Overall, our testing through simulation-optimization suggests that both the models can represent the dynamic behaviors of the demand sites with an acceptable degree of accuracy.

### Dynamic behavior of reservoirs

**Fig 8:**
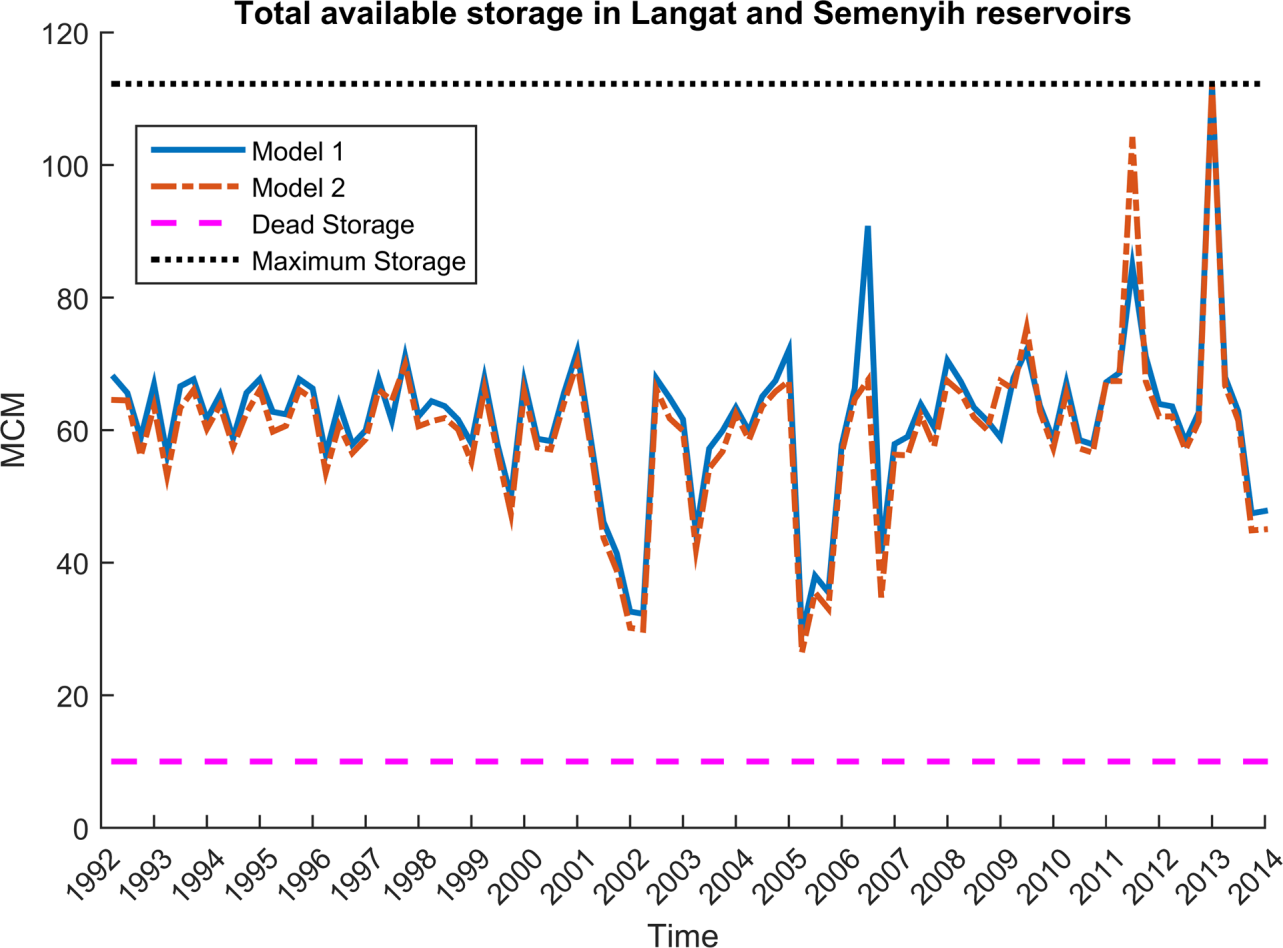
Comparision between models in terms of storage.

Fig 8 below shows the total reservoir storage for both models during the simulation-optimization process. As can be seen, the first model tends to keep more water in the reservoirs. It displays less flexibility as it plays a pure strategy game, leading to less release of water during drought events as well as less storage of water in high rainfall or flood situations. In contrast, the second model adjusts its behavior more flexibly and dynamically based on the expected situation in the next time step. This model thus allows the release of more water during drought events (2002, 2005, 2007), reducing the unmet demand of demand sites. It also generally creates higher storage levels during high flow situations to protect the downstream from flooding. At the same time, where there was a high risk of flooding in the next step, the second model reduced the storage level–to avoid a failure (flooding) like the one that actually occurred in the second season of 2006. In conclusion, employing a combination of the Markov chain method and mixed-strategy game undoubtedly increased the performance of the reservoirs.

Table 1 below contains an evaluation of the reliability of the two models. As can be seen, both the seasonal volumetric and occurrence reliability indices were relatively high for both models. The demand site indices for the second season, a wet season, were higher (better) than for the other seasons–although there were still some minor failures in this season, mainly because the models saved up water for dry seasons. The fourth season, also a wet season, meanwhile produced the lowest indices as a result of flooding events.

These results suggest that, overall, the two models performed roughly equally well in terms of the reservoir performance indices, albeit the seasonal performance indicators of the demand sites were slightly better for the first model.

**Table 1:** Seasonal performance of models.

	Inside Basin*	Outside Basin*	Langat Dam	Semenyih Dam
	Occurrence Reliability			
Season 1				
Model 1	86.4	86.4	100	100
Model 2	81.8	86.4	100	100
Season 2				
Model 1	90.9	90.9	100	100
Model 2	81.8	86.4	100	100
Season 3				
Model 1	77.3	77.3	100	100
Model 2	72.7	77.3	100	100
Season 4				
Model 1	90.9	90.9	95.5	95.5
Model 2	72.7	81.8	95.5	95.5
	Volumetric Reliability			
Season 1				
Model 1	96.8	94.9	0	0
Model 2	96.7	94.8	0	0
Season 2				
Model 1	98.8	98	0	0
Model 2	98.5	98	0	0
Season 3				
Model 1	96.7	94.3	0	0
Model 2	96.4	94	0	0
Season 4				
Model 1	98.3	97.1	0.018	0.005
Model 2	98	97.1	0.017	0.005

* Demand site

**Fig 9:**
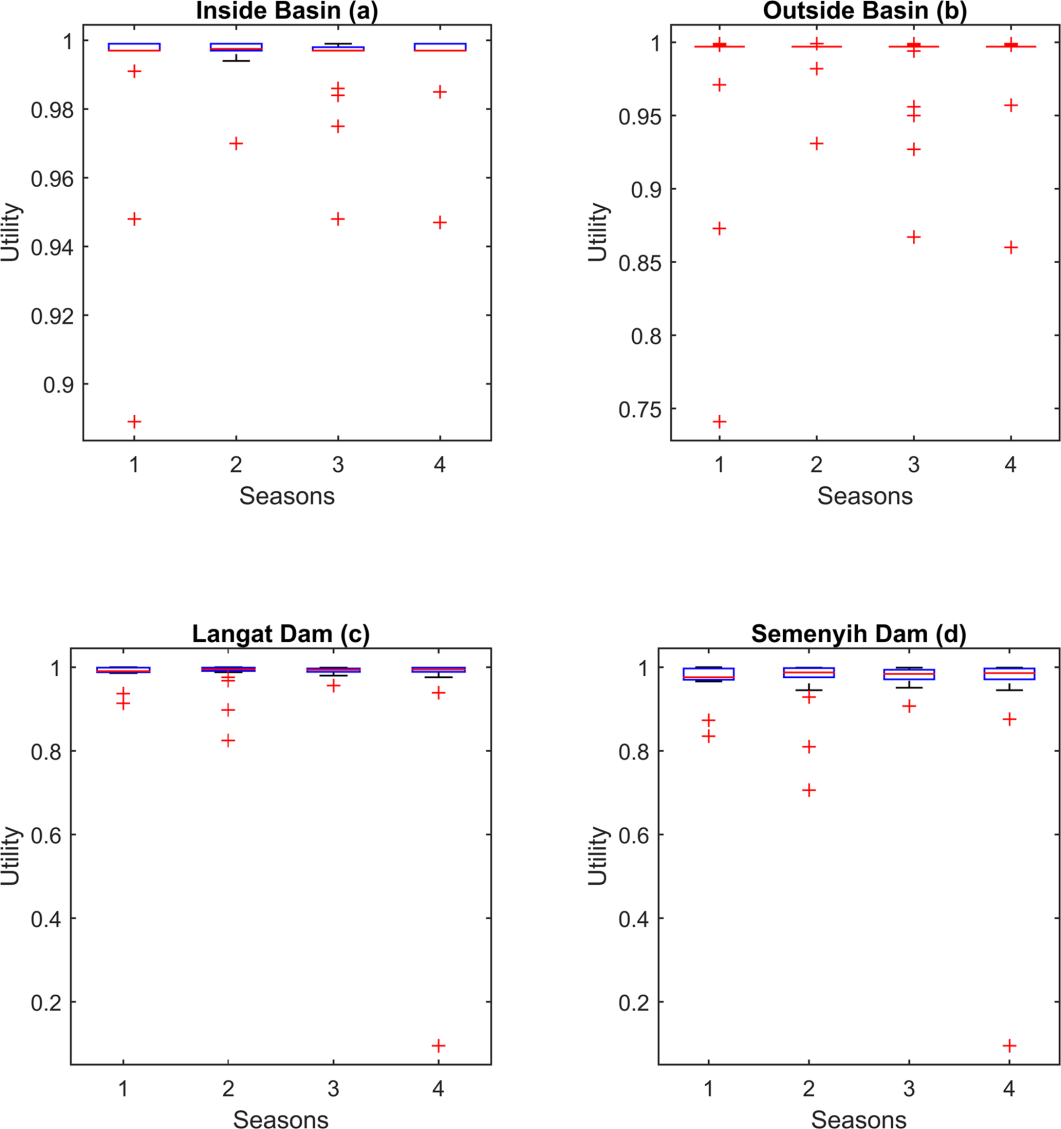
Seasonal utilities of components resulting from simultaneous simulation and optimization of the models.

Fig 9 illustrates the utilities resulting from the simulation-optimization process. In terms of overall water player satisfaction (utility), both models again produced broadly similar results. The difference between them with regard to the level of demand site satisfaction was negligible. However, reservoir operator satisfaction was slightly lower for the second model–presumably due to the tendency of the latter to keep less water in the reservoirs. With both models, the satisfaction levels of outside basin demand site fell more compared with the first model during drought events, no doubt as a result of increased water demand. Specifically, satisfaction fell below 75% for the outside basin demand site in the worst drought event in 2005, while it remained higher, at just under 90%, for the inside basin demand site. Finally, reservoir operator satisfaction fell to its lowest level– 0%–due to the serious flooding event in 2013, when both reservoirs were completely full and unable to cope with further water flows.

### Overall comparison between presented models

In this study, we developed two seasonal continuous dynamic models to tackle water allocation conflicts in a complicated water system comprising demand sites, river tributaries (uncontrolled water) and reservoirs (controlled water). The first model demonstrated the application of system dynamics components coupled with a Nash bargaining solution optimizer, giving it the capability to optimize reservoir storage and demand site allocations simultaneously. The second model successfully added a combination of the mixed-strategy game and Markov chain methods to adjust reservoir management further in order to take account of uncertainty in hydrological inputs. This model not only took account of the internal dynamic behaviors of demand site components but also calculated the optimal release levels of water for each season based on expected inflows for the next one, so as to optimize the overall performance of the system.

Table 2 summarizes the capabilities and overall performance of both models. The overall satisfaction and reliability indices for the demand sites were marginally better for the first model. On the other hand, applying the coupled Markov chain and mixed-strategy game method in our second model did produce a noticeable improvement in the vulnerability ratings of the reservoirs over the first model. Moreover, not only vulnerability but also resilience were slightly higher for the inside basin demand site in the second model. Resilience improved from 45% to 50%, while vulnerability fell from 11.218 MCM to 9.993 MCM. However, the application of our second model had a slight negative effect on the vulnerability of outside basin demand sites.

In this context, it should be noted that the seasonal hydrological sequence in the Langat River basin is *dry-wet-dry-wet*. During the wet seasons, inflows are normally higher than demand, and there is sufficient water available to fill up the reservoirs. This means that the second model needs to predict only one step ahead in order to deal with inflow uncertainty–which makes the Markov chain an appropriate method to utilize. Moreover, the application of complex models for more complicated situations or catchments usually requires large amounts of very detailed data; hence also our choice of a relatively simple model for this study to promote the advantages of combining game theory and system dynamics.

**Table 2:** Overall performance of the models.

Model	Model 1	Model 2
Model proficiency		
Allocation Optimization	✓	✓
Storage Optimization	✓	✓
Applied game strategies	Pure	Mixed
Overall Satisfaction		
Demand Points Ave. Satisfaction (%)	99.2	99.1
Reservoirs Ave. Satisfaction (%)	97.1	92.3
Model Ave. Satisfaction (%)	98.1	95.7
Occurrence Reliability		
Inside Basin Demand Site	87.5	86.4
Outside Basin Demand Site	86.4	86.4
Langat Reservoir	98.9	98.9
Semenyih Reservoir	98.9	98.9
Volumetric Reliability		
Inside Basin Demand Site	97.7	97.5
Outside Basin Demand Site	96.1	96
Langat Reservoir	0.005	0.004
Semenyih Reservoir	0.001	0.001
Resilience		
Inside Basin Demand Site	45.5	50
Outside Basin Demand Site	50	50
Langat Reservoir	100	100
Semenyih Reservoir	100	100
Vulnerability		
Inside Basin Demand Site	11.218	9.993
Outside Basin Demand Site	27.95	28.672
Langat Reservoir	9.14	8.62
Semenyih Reservoir	4.66	4.05

## Conclusion

In this study, we developed and applied a combination of system dynamics and game theory as a simulation-optimization method for water conflict resolution. This method benefits both from the feedback loops and certain other advantages of system dynamics, as well as the ability of game theory to help identify optimized decisions based on preferences. An additional advantage of this method is that it is relatively easy to apply to integrated water resource management, since it incorporates the preferences of all stakeholders and decision makers into the decision-making process, takes account of the dynamicity of the system, and is easily extendable to multi-reservoir systems using object-oriented programming concepts.

This study shows that the suggested method, combining system dynamics and game theory, is highly capable of managing available water resources and resolving conflicts over water allocation. Both our models–the first based on pure game strategy and the second incorporating mixed game strategy–produced broadly similar strong performances in managing water allocation between the demand sites. While the first model scored slightly higher on some indices of satisfaction and reliability, the combined application of the mixed strategy game and Markov chain methods produced noticeably better results for resilience and vulnerability, making the second model the overall better performer.

## Supporting information

S1 Table The inflow data used as input for the models. (XLSX)

S2 Table The Ln(GDP) used as input for the models. (XLSX)

S3 Table The results of the first model. (XLSX)

S4 Table The results of the second model. (XLSX)
